# Mechanisms of cisplatin sensitivity and resistance in testicular germ cell tumors

**DOI:** 10.20517/cdr.2019.19

**Published:** 2019-09-19

**Authors:** Ratnakar Singh, Zeeshan Fazal, Sarah J. Freemantle, Michael J. Spinella

**Affiliations:** 1 Department of Comparative Biosciences, University of Illinois at Urbana-Champaign, Urbana, IL 61801, USA.; 2 The Carle Illinois College of Medicine , University of Illinois at Urbana-Champaign, Urbana, IL 61801, USA.; 3 The Cancer Center of Illinois, University of Illinois at Urbana-Champaign, Urbana, IL 61801, USA.

**Keywords:** Testicular cancer, embryonal carcinoma, cisplatin, resistance, epigenetics, testicular germ cell tumors, DNA methylation, p53

## Abstract

Testicular germ cell tumors (TGCTs) are a cancer pharmacology success story with a majority of patients cured even in the highly advanced and metastatic setting. Successful treatment of TGCTs is primarily due to the exquisite responsiveness of this solid tumor to cisplatin-based therapy. However, a significant percentage of patients are, or become, refractory to cisplatin and die from progressive disease. Mechanisms for both clinical hypersensitivity and resistance have largely remained a mystery despite the promise of applying lessons to the majority of solid tumors that are not curable in the metastatic setting. Recently, this promise has been heightened by the realization that distinct (and perhaps pharmacologically replicable) epigenetic states, rather than fixed genetic alterations, may play dominant roles in not only TGCT etiology and progression but also their curability with conventional chemotherapies. In this review, it discusses potential mechanisms of TGCT cisplatin sensitivity and resistance to conventional chemotherapeutics.

## INTRODUCTION

Testicular germ cell tumors (TGCTs) are the most common neoplasm in men age 15 to 44 years old with increasing incidence in the last 40 years^[1]^. Metastatic TGCTs have been a model for transforming a once fatal metastatic solid tumor into one that is curable. The discovery in the mid-70s that TGCTs are sensitive to conventional cisplatin-based combination chemotherapy eventually resulted in an improvement in 5-year survival from less than 10% to over 80% even in patients with disseminated disease^[2,3]^. However, 15%−20% of all patients and 50% of poor risk patients are refractory to treatment and only 50% of patients can be cured after relapse with salvage therapy consisting of high dose platinum followed by stem cell transplant or traditional chemotherapies^[4–6]^. Most patients progressing after high dose therapy will succumb to their disease as will those patients who undergo late relapse^[6,7]^. Attempts to incorporate newer targeted therapies to treat refractory TGCTs have been unsuccessful. An average of nearly 40 years of life are lost when a patient dies from testicular cancer, over a decade more than any other adult malignancy^[8,9]^. Further, TGCT patients successfully treated with cisplatin-based therapies suffer from acute and life-long toxicities including infertility, hypogonadism, androgen deficiency, decreased lung and kidney function and neurotoxicity and have a greater risk of developing cardiovascular disease and secondary malignancies^[10]^. Hence, there is a pressing clinical need to devise new strategies to treat cisplatin refractory TGCTs and a rationale to devise targeted, cisplatin-sparing therapies.

TGCTs remain the only solid malignancy curable with chemotherapy. A greater understanding of the hypersensitivity and resistance of TGCTs has the potential to not only impact refractory patients but also may inform strategies to sensitize other solid tumors to conventional chemotherapies^[11,12]^. TGCTs are classified based on histology into two distinct subtypes seminomas and nonseminomas. Both seminomas and nonseminomas likely arise from precursor cells called germ cell neoplasia in situ (GCNIS)^[13–15]^. Nonseminomas can be further classified as embryonal carcinoma (EC), teratoma, yolk sac tumor and choriocarcinoma. Pluripotent EC are the stem cell-like component of nonseminoma. TGCTs and EC have an intrinsic hypersensitivity to drug-induced cell death^[16,17]^. The basis of this hypersensitivity and mechanisms to account for chemotherapy resistance remain elusive. TGCTs likely represent transformed germ cells and may have inherited unique mechanisms of sensitivity to DNA damage and other stress to prevent germline mutations. Alterations in traditional mechanisms of cisplatin sensitivity and resistance in other solid tumors have generally not been accepted as the reasons for the hypersensitivity of TGCTs^[3,12,18]^.

TGCTs have one of the lowest overall somatic mutation rates of all solid tumors and possess unique epigenetic states that are likely a reflection of the epigenetics of their primordial germ cell (PGC) origins^[19,20]^. Further EC and other pluripotent cells are known to undergo extensive plasticity in tumorigenicity depending on the environment^[21,22]^. These findings suggest that epigenetics may play a larger role in the chemotherapeutic hypersensitivity and resistance of TGCTs. Recently genetic susceptibility, biological signaling, and genetic and environmental factors have been examined to explain the mechanisms responsible for TGCTs curability, pathogenesis and development of treatment resistance. This review will focus on potential mechanisms of cisplatin resistance in TGCTs that may relate to the exquisite sensitivity of this solid tumor to conventional chemotherapeutics that may provide strategies to overcome acquired cisplatin resistance.

## ETIOLOGY AND PATHOLOGY OF TGCTS

**Figure 1:**
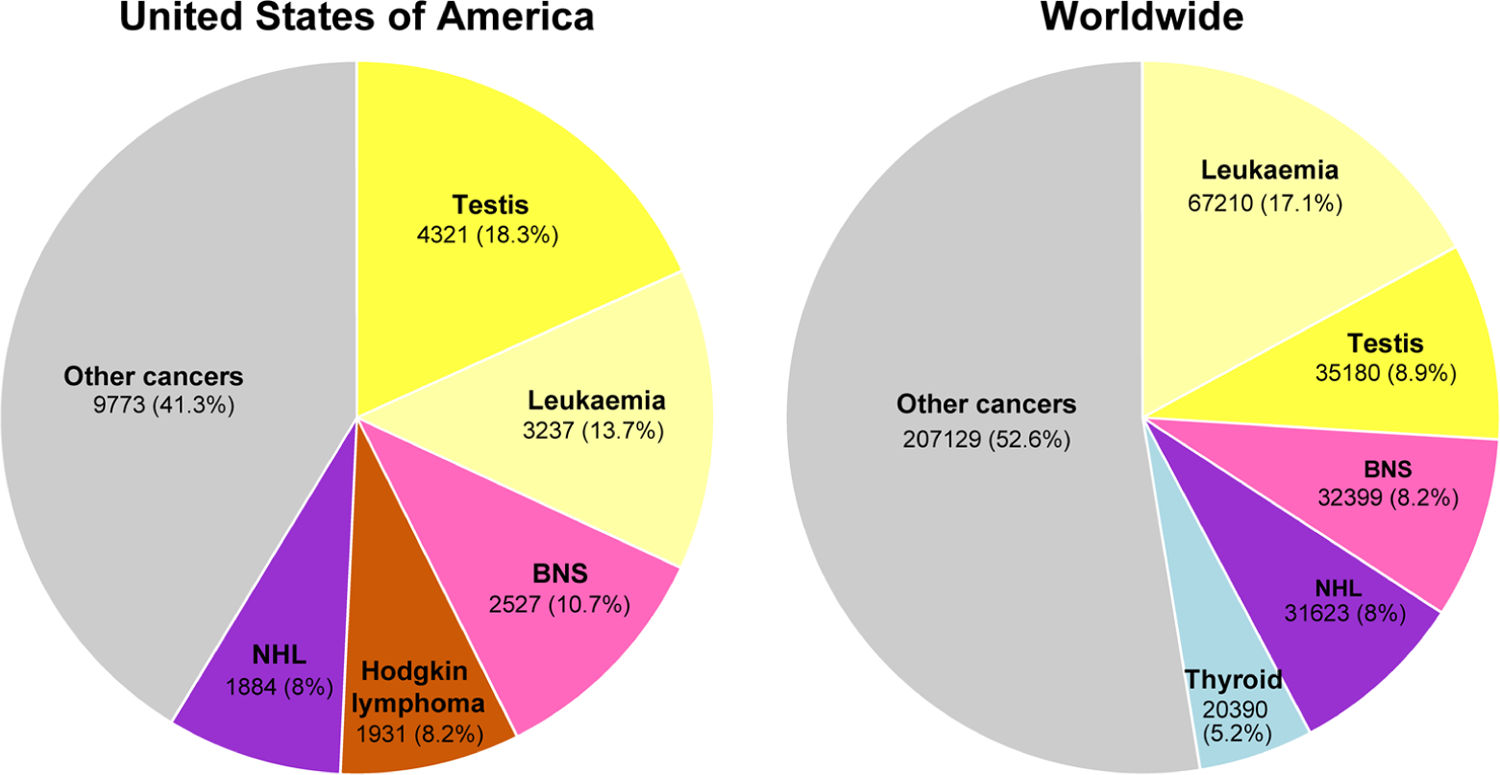
Estimated new cancers in males aged 0–34 in the USA and worldwide for 2018. Pie charts represent the distribution of new cancer cases in the United States and worldwide for males 0–34 years of age. Data source: World Health Organization global cancer observatory.

**Figure 2:**
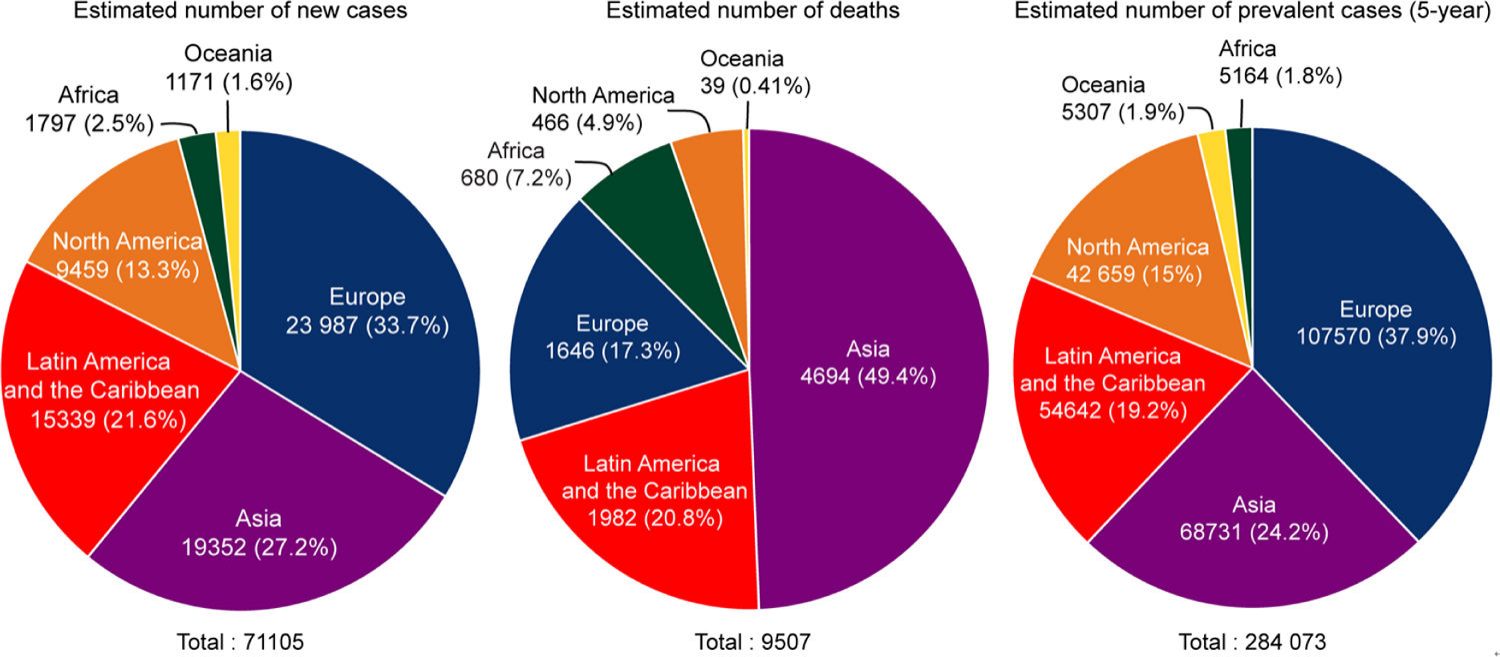
Estimated number of new TGCT cases, deaths and prevalence worldwide for 2018. Pie charts represent the distribution of new TGCT cases, TGCT deaths and TGCT prevalence worldwide for all aged males. Data source: WHO global cancer observatory. TGCTs: testicular germ cell tumors

The incidence of TGCTs has been steadily rising in young males^[9]^. According to the 2018 WHO global cancer observatory, TGCTs are estimated to have the largest number of new cases among all cancers for males under the age of 34 years in the USA and second largest worldwide [Figure 1]^[23]^. The incidence of TGCTs varies widely with geographic location with the highest incidence in northern European countries and the lowest in Africa nations [Figure 2]. The reasons for these differences is not known but is hypothesized to be a combination of inheritable and environmental factors including exposure to endocrine disruptors *in utero*. Common risks for TGCTs include endocrine disruptors, cryptorchidism^[24]^, Down’s syndrome^[25]^, family history of cancer^[26]^, low birth weight, premature birth, birth order, bleeding during pregnancy, hormone exposure during pregnancy, high maternal age and neonatal jaundice^[27,28]^. Late age at puberty, male infertility and testicular dysgenesis syndrome are also associated with TGCTs^[29–33]^.

**Figure 3:**
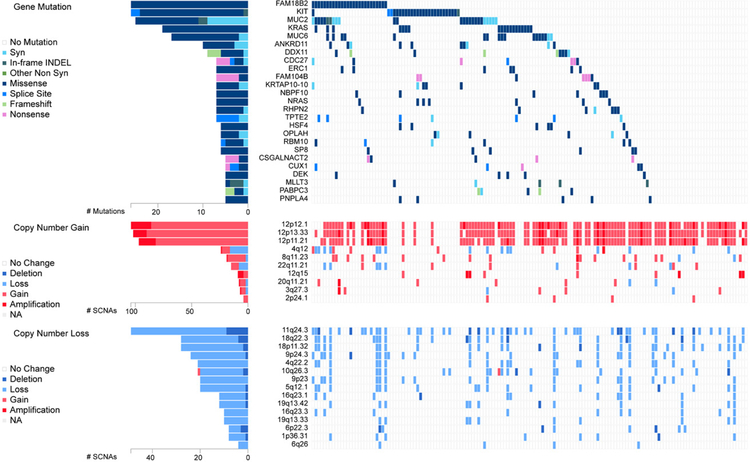
Genetic alteration in TGCTs. Analysis of 150 patient TGCT cohort generated through FireBrowse. Top genetic alterations are arranged by frequency. Data source: TCGA. TGCTs: testicular germ cell tumors

EC cells resemble embryonic stem (ES) cells^[22]^. Both cell types possess pluripotency and have close similarities in gene expression^[34,35]^. Interestingly, despite similarities, EC cells are malignant, while ES cells are not. GCNIS-derived TGCTs have cytogenetic and molecular anomalies that mainly include aneuploidy and gain and loss of distinct chromosomal regions coupled with somatic mutation rates that are low^[19,20]^. Chromosome 12p amplification, such as isochromosome 12p and chromosome 12p overrepresentation are nearly universally present and pathognomonic for TGCTs^[36,37]^. Other chromosomal anomalies associated with TGCTs include gain of genetic material on chromosome 1, 2p, 7, 8, 12, 14q, 15q, 17q, 21q, and X and the deletion of genetic material from chromosome 4, 5, 11q, 13q, and 18q2^[38]^. Similarly, multiple passaging of ES cells has been shown to result in acquired alterations similar to those found in TGCTs and EC, including chromosome 12, 17, and X gain^[39]^. A recent analysis performed on a 150 TGCT cohort from the TCGA^[40]^ indicates gain/amplification of 12p in the majority of patients with gain in 12q, 8q, 22q and deletion/loss of 11q, 18q, 18p, 9p, 4q, 10q, 5q, 16q and 19q also occurring with much less frequency [Figure 3].

The GCNIS legion is considered a precursor for all TGCT subtypes. GCNIS has been hypothesized to emerge from PGCs or early gonocytes that have transformed *in utero*^[41–43]^. Evidence that TGCTs begin during development include similarities in expression of the markers KIT, OCT4, and PLAP, cell morphology, genomic imprinting and transcriptomics between GCNIS and PGCs^[44,45]^. Further, abnormalities of germ cell development are associated with TGCT incidence^[46]^. In addition extragonadal TGCTs are found at the midline, which is where PGCs migrate during development^[47,48]^. Taken together the data support that TGCTs come from arrested PGCs or gonocytes that lie dormant but further progress with gonadal hormones during sexual maturation^[41]^.

Genetic drivers of TGCTs are poorly understood compared to other cancers. Several genes have been implicated in the pathogenesis of TGCTs, but their exact roles are still uncertain and underscore that TGCT etiology is likely widely polygenic in nature. Genome-wide association studies have been useful in identifying variants that contribute to risk of TGCTs. Allelic variation within the kit-ligand gene 12q22 is the strongest genetic risk factor for TGCTs^[49]^. Several other allelic variants have been identified that correlate with TGCTs susceptibility^[45]^. Somatic mutation in TGCTs are relatively rare and of relatively low frequency and occur mostly in seminoma. Gain-of function mutations in KIT are most frequent at 18%^[19,20]^. Other mutations in seminoma include KRAS (14%) and NRAS (4%) [Figure 3].

## EPIGENETICS OF TGCTS

There is considerable evidence that epigenetics may play a role in TGCT biology. As stated above, environmental exposures *in utero* and testicular dysgenesis syndromes are major risk factors for TGCTs. Further, normal ES cells are very similar in gene expression and biology to EC cells. Transformation of both of these pluripotent cell types are influenced by environmental factors exemplified by findings that ES cells form teratoma and teratocarcinomas when implanted ectopically in mice, while EC cells in certain situations can participate in normal development when placed in the early embryo^[21,50]^. Recent studies have characterized the pattern of TGCT DNA methylation. It is important to remember that PGCs normally undergo erasure of imprinting during development^[51]^. Thus, TGCTs may have arisen from a germ cell lacking imprinting. Evidence to support this comes from the finding that IGF2 and H19 are expressed from both alleles in TGCTs but are otherwise exclusively expressed from one parental allele in adult tissues^[52]^. TGCT genomic DNA is hypomethylated in comparison to most solid tumors. Seminomas possess very little DNA methylation while EC have an intermediate level of DNA methylation compared to somatic cancers and EC-derived teratoma are hypermethylated^[20,52–55]^. The level of DNA methylation roughly correlates with cisplatin curability with seminoma being the most curable followed by EC and other nonseminoma components besides teratoma. Teratoma is resistant to cisplatin treatment.

Interestingly, pluripotent cells including EC cells possess high levels of non-CpG methylation called mCpH (H = A, C, T) along gene bodies of genes that are highly expressed^[52,56]^. In EC up to 25% of all cytosine methylation is mCpH while over 99% of all cytosine methylation in somatic cells is CpG. Also, mCpH does not occur in the mature components of nonseminoma or in seminoma, suggesting mCpH is closely associated with the pluripotent and reprogrammed state of EC^[52]^. The role of non-CpG methylation in EC is currently unclear.

Histone modifications are well-established epigenetic regulators. Polycomb complexes modify chromatin to repress homeotic genes which have an important role in stem cell dynamics^[57,58]^. Gene repression is initiated by polycomb repression complex 2 which contains histone methyltransferase activity resulting in histone H3 trimethylation on lysine 27 (H3K27me3). The H3K27me3 then recruits PRC complex 1 to further mediate repression of gene expression. Alterations in BMI1 and EZH2 and other polycomb components frequently occurs in cancer^[59,60]^. Little is known concerning alterations in histone modifiers in TGCTs^[61,62]^. The H3K4me demethylase, LSD1 has been shown to be a key regulator of pluripotency in ES and EC cells. LSD1 has been reported to be highly expressed in EC and seminoma which correlates with LSD1 and HDAC1 inhibitor sensitivity^[63,64]^.

Another form of epigenetic regulation recently studied in TGCTs is mediated by miRNAs. Voorhoeve *et al*.^[65]^, showed that in TGCT cells miR-372 and miR-373 neutralized p53 function through inhibition of the tumor suppressor LATS2. Several miRNAs appear to be specifically expressed in TGCTs and have been investigated as potential new serum biomarkers for TGCT patients^[66]^. For example, miR-371a-3p was reported to have high sensitivity and specificity for detecting TGCT disease burden and was not elevated in control patients or patients with other cancer types^[66,67]^. Serum levels of miR-371a-3p increased with TGCT progression and decline after surgery^[66,67]^. Since TGCTs appear to have distinct epigenetics compared to normal tissues and other cancers, the use of epigenetic biomarkers may be particularly useful for disease management of TGCT patients.

## MECHANISM OF CISPLATIN RESISTANCE IN TGCTS

Several principles of solid tumor chemotherapy were validated during the optimization of curative TGCTs regimens. Seminomas are very sensitive to cisplatin-based therapy and are highly curable regardless of stage^[2]^. Nonseminoma are somewhat less sensitive to chemotherapy especially for tumors of more advanced grade and stage^[2]^. For advanced TGCTs, bleomycin, etoposide and cisplatin or etoposide, ifosfamide and cisplatin (VIP) are the first line standards of care. The International Germ Cell Cancer Collaborative Group devised a risk classification based on serum markers and primary and metastatic sites that divided metastatic nonseminoma patients into good, intermediate, and poor risk with cure rates of 90%, 75% and 50%, respectively^[68]^. Currently there are no predictive biomarkers to identify which poor-risk patients will die from disease. There is a clinical need to better identify which high risk patients will fail conventional therapy. Salvage high-dose chemotherapy with stem cell rescue or conventional dose chemotherapy can induce remissions in approximately 25% of relapse cases^[69]^. The treatment of refractory multi-relapsing disease is very challenging and most cases cannot be cured. Targeted approaches including tyrosine-kinase inhibitors and immunotherapy have failed to demonstrate activity in these refractory cases. Mechanisms of hypersensitivity and acquired resistance in TGCTs are likely multi-factorial and could include cellular detoxification, altered platinum accumulation, DNA repair, and alterations in apoptotic pathways^[12,18,70]^. However, these mechanisms are shared by many solid tumors that are not curable. Further TGCTs are highly sensitive to a variety of agents that do not share common import and export pathways and mediate different forms of DNA damage repaired by distinct mechanisms. Hence, mechanisms responsible for TGCT sensitivity and resistance may be related to how these tumors respond to damaged DNA mediated in part by their unique cellular context^[3,71]^. In the next section, we will review suggested mechanisms of therapy resistance in TGCTs.

**Figure 4:**
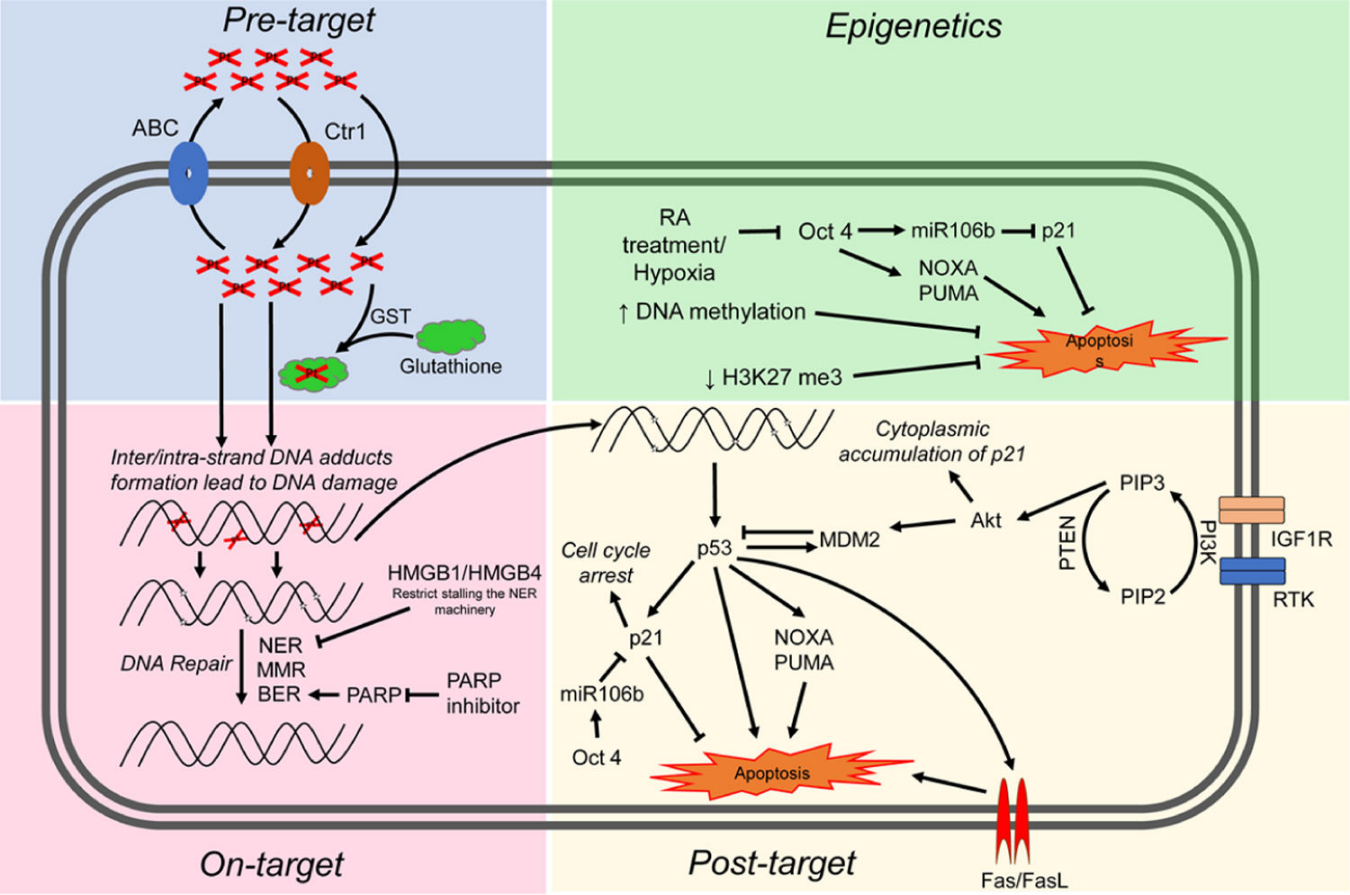
Proposed mechanisms mediating cisplatin sensitivity and resistance in TGCTs. Cisplatin resistance mechanisms are classified as pre-target, on-target, post-target and epigenetic mechanisms. ABC: atp-binding cassette transporters; GST: glutathione s-transferase; NER: nucleotide excision repair; MMR: mismatch repair; BER: base excision repair; RA: retinoic acid; RTK: receptor tyrosine kinase; TGCTs: testicular germ cell tumors

Cisplatin resistance mechanisms can be classified as pre-target, on-target, and post-target^[70]^. Pre-target involve alterations preceding the binding of cisplatin to DNA, on-target resistance involves alterations directly relate to DNA-cisplatin adducts, post-target resistance involves mechanisms downstream of cisplatin-mediated DNA damage [Figure 4]. In this section we will discuss these cisplatin resistance mechanisms.

### Pre-target resistance

Before cisplatin binds DNA, cancer cells can avoid cytotoxicity by two main mechanisms: first, decreased cellular accumulation of cisplatin and second, enhanced cisplatin detoxification by glutathione, metallothioneins and nucleophilic cytoplasmic “scavengers”^[70]^. Cellular uptake of cisplatin is typically mediated by passive diffusion or facilitated transport, although the copper transporter SLC31A1 has also been implicated as a cisplatin transport mechanism^[72]^. Cisplatin uptake rates in TGCT and other cancer cells have been reported to be similar^[73]^. A number of ATP-binding cassette transporters including MDR1 have been linked to cisplatin efflux^[70]^. However, no cisplatin resistance mechanism related to drug transporters has been reported for TGCTs.

Resistance to cisplatin is often associated with increased levels of thiol-containing proteins such as glutathione and metallothionein which detoxify cisplatin through conjugation. In general it appears that the level of these detoxifiers are low in TGCTs compared to some other tumor types^[74]^. Interestingly, one study showed high levels of a glutathione-S-transferase, an enzyme that conjugates GSH in resistant teratoma^[75]^. These finding suggest that low levels of exporters and detoxifiers may play at least a contributory role in TGCT sensitivity to cisplatin. However, in general pre-target mechanisms have not been widely recognized as significant sources of cisplatin resistance in TGCTs.

### On-target resistance

Inter/intra-strand DNA adducts and subsequent apoptosis can be defective in cisplatin-resistant cancer cells due to a variety of mechanisms. TGCTs have been shown to have similar rates of cisplatin DNA adduct formation upon initial exposure compared to other tumor types^[71,74,76]^. However, other reports have implied that TGCTs possess a diminished capacity to repair cisplatin adducts^[77,78]^. Correspondingly, cisplatin-resistant cells may have acquired the ability to repair adducts at an enhanced rate or gained the ability to tolerate unrepaired lesions. The majority of cisplatin DNA lesions are repaired by the nucleotide excision repair (NER) system^[79,80]^. One proposed mechanism for cisplatin resistance involves high expression of the high-mobility group box protein 1 (HMGB1) in TGCT cells^[81]^. HMGB1 appears to bind selectively to cisplatin DNA crosslinks and interferes with NER^[82–85]^. Similar to HMGB1, HMGB4 is also highly expressed in TGCTs and likewise recognizes cisplatin-DNA intrastrand cross-links, stalling the NER machinery which otherwise would excise and repair the damage^[81]^. Hence, one proposed mechanism to explain the chemosensitivity of TGCT is decreased capacity to repair interstrand platinum-DNA adducts, the most toxic from of cisplatin induced adducts by either excision repair or homologous recombination. We refer the reader to an excellent recent review on this topic^[86]^.

Several studies have linked defective mismatch repair (MMR), microsatellite instability and BRAF mutations with relapse and treatment failure in TGCT patients^[87–90]^. Refractory TGCTs have been shown to have decreased expression of MMR genes including MLH1, MLH2, or MSH6, suggesting that decreased MMR may be a mechanism of TGCT resistance^[89]^. However, the precise molecular mechanisms are unclear. Future studies are needed to solidify the exact role of MMR in cisplatin resistance in TGCTs. Poly (ADP-ribose) polymerase (PARP) is involved in the repair of DNA single-strand breaks via base excision repair (BER), a key cellular DNA repair pathway. Mego and colleagues observed overexpression of PARP in TGCTs compared to normal testicular tissue and PARP inhibitors were shown to reverse cisplatin resistance^[91,92]^.

### Post-target resistance

Post-target resistance to cisplatin can result from alterations in signal transduction pathways that mediate apoptosis in response to DNA damage. Non-repairable cisplatin-induced DNA damage leads to the activation of a multi-branched signaling cascade with proapoptotic outcomes. A distinguishing feature of TGCTs is that they are devoid of p53 mutations compared to almost all other solid tumors^[19,20]^. Several groups have provided evidence that hyperactivation of p53 is a key mechanism for the hypersensitivity of TGCTs to cisplatin^[93–97]^. However, the role of p53 mutations in cisplatin resistance of TGCTs has been controversial^[3,96]^. Mutations in p53 and overexpression of MDM2 do appear to occur in a proportion of cisplatin refractory TGCTs but the extent of these mutations is unclear and will require studies with larger numbers of cisplatin refractory cases^[98,99]^. High abundance of MDM2 has been shown to correlate with cisplatin resistance in TGCT cell lines and disruption of the MDM2-p53 interaction by nutlin-3 strongly potentiated p53-dependent apoptosis via the Fas/FasL pathway^[100]^. NOXA and PUMA, downstream targets of p53 and p53 family members p63 and p73, have been linked to the cisplatin sensitivity of TGCTs^[95,101,102]^. Overexpression of the cell cycle regulator CCND1 has been described as a mechanism of cisplatin resistance^[103]^. Upregulation of IGF1R expression and signaling was also found to contribute to acquired cisplatin resistance in an *in vitro* nonseminoma model^[104]^.

Di Vizio *et al*.^[105]^ provided evidence that the PDGFR/PI3K/AKT pathway plays a role in the development of TGCTs. Dysregulation was mainly due to loss of PTEN in TGCTs. Other studies demonstrated a role for this pathway in cisplatin resistance in TGCTs patients and cell lines^[106,107]^. Inhibition of PDGFRβ mediated phosphorylation of AKT by Ly294002 reversed cisplatin resistance in TGCT cell lines^[107]^. Furthermore, activation of the PI3K/AKT pathway phosphorylated MDM2 and p21 which led to cytoplasmic accumulation of p21 and inhibition of p53 mediated apoptosis in response to cisplatin^[106,107]^. Somatic mutations within PI3KCA, AKT and FGFR3 were also reported in cisplatin resistant TGCTs^[108]^. Taken together, pre-clinical studies indicate that targeting this pathway may reverse cisplatin resistance in TGCTs^[107,109,110]^. However, Mego *et al*.^[111]^ showed limited efficacy of the mTOR inhibitor everolimus against unselected heavily pretreated refractory TGCTs.

The cisplatin-resistant phenotype can also be sustained by alterations in signaling pathways that are not directly engaged by cisplatin, so called “off-target” effects^[112–114]^. It is well recognized that epigenetic alterations occurring upon differentiation increase cisplatin resistance in TGCT cells^[115]^. TGCTs have been shown to undergo differentiation after retinoic acid treatment and molecular changes that occur in these cells after treatment have been well characterized^[114,116]^. Gutekunst *et al*.^[95]^ demonstrated short-term differentiation of EC cells by retinoic acid significantly decreases the expression of IER3, NOXA and PUMA and caused a loss in cisplatin hypersensitivity. Other studies have reported that loss of OCT4 expression leads to cisplatin resistance in EC cells^[112,113]^. Events in addition to differentiation that can lead to downregulation of OCT4 include hypoxia^[117]^ and cisplatin treatment^[118]^. OCT4 is also known to induce the expression of miR106b which in turn suppresses p21 expression^[106]^. Similarly, increased OCT4 expression was also significantly correlated with cisplatin response in xenograft TGCTs models, cell lines, and tumors^[112,116,119,120]^. Altogether, these finding suggest a connection between OCT4, NOXA and the cytoplasmic expression of p21 in cisplatin resistance TGCTs.

### Epigenetics

Epigenetic silencing, for example linked to hypermethylation, could mediate TGCT cisplatin resistance by both on-target and post-target/off target mechanisms. Accumulating evidence suggests that an increase in DNA methylation may be associated with cisplatin resistance^[61,121,122]^. Seminomas are generally considered more sensitive to cisplatin than nonseminoma and are severely hypomethylated. EC cells are also sensitive to cisplatin but have a higher incidence of resistance and have an intermediate level of methylation while more difficult to treat teratomas, yolk sac tumors and choriocarcinoma have the highest level of DNA methylation^[37,52]^. Methylation of several specific gene promoters have been associated with inherent and acquired cisplatin resistance in TGCTs and cell lines including RASSF1A, HIC1, MGMT and CALCA^[123–125]^. Additionally, cisplatin treatment has been shown to be associated with increased DNA methylation *in vivo*^[123]^. We have shown that a series of testicular cancer derived EC cell lines are hypersensitive to the DNA methytransferase inhibitors decitabine and quadecitabine *in vitro* and *in vivo* compared to somatic tumors^[126–128]^. This hypersensitivity extended to cisplatin refractory EC and was dependent on high levels of the DNA methyltransferase, DNMT3B. Importantly, pretreatment of cisplatin refractory cells *in vitro* and *in vivo* could resensitize them to cisplatin and this activity was associated with activation of p53 and immune-related pathways^[126–128]^. These findings have been validated by a number of additional studies^[124,129,130]^. This data provided the rationale to combine cisplatin and guadecitabine in a phase I study at Indiana University ]. Significant responses were observed including two complete and two partial remissions in heavily pretreated platinum refractory TGCT patients (Spinella *et al*., in preparation). In a new study with an extended series of cisplatin resistant EC cells we unexpectedly found a tight association between cisplatin resistance and decreased H3K27 methylation of polycomb target genes that was associated with a decrease in expression of BMI1 and EZH2^[131]^ (Spinella *et al*., in preparation). How the decrease in H3K27 methylation of polycomb targets relates to DNA methylation is currently being investigated. Interestingly, decreased H3K27 methylation has been previously associated with increased DNA promoter methylation in ES cells^[132–134]^. Another promising approach to overcome cisplatin resistance in a number of cancer types including TGCTs is the used of the bromodomain inhibitor JQ1 that target the epigenetic reader bromo and extra-terminal domain proteins^[135–137]^.

**Figure 5:**
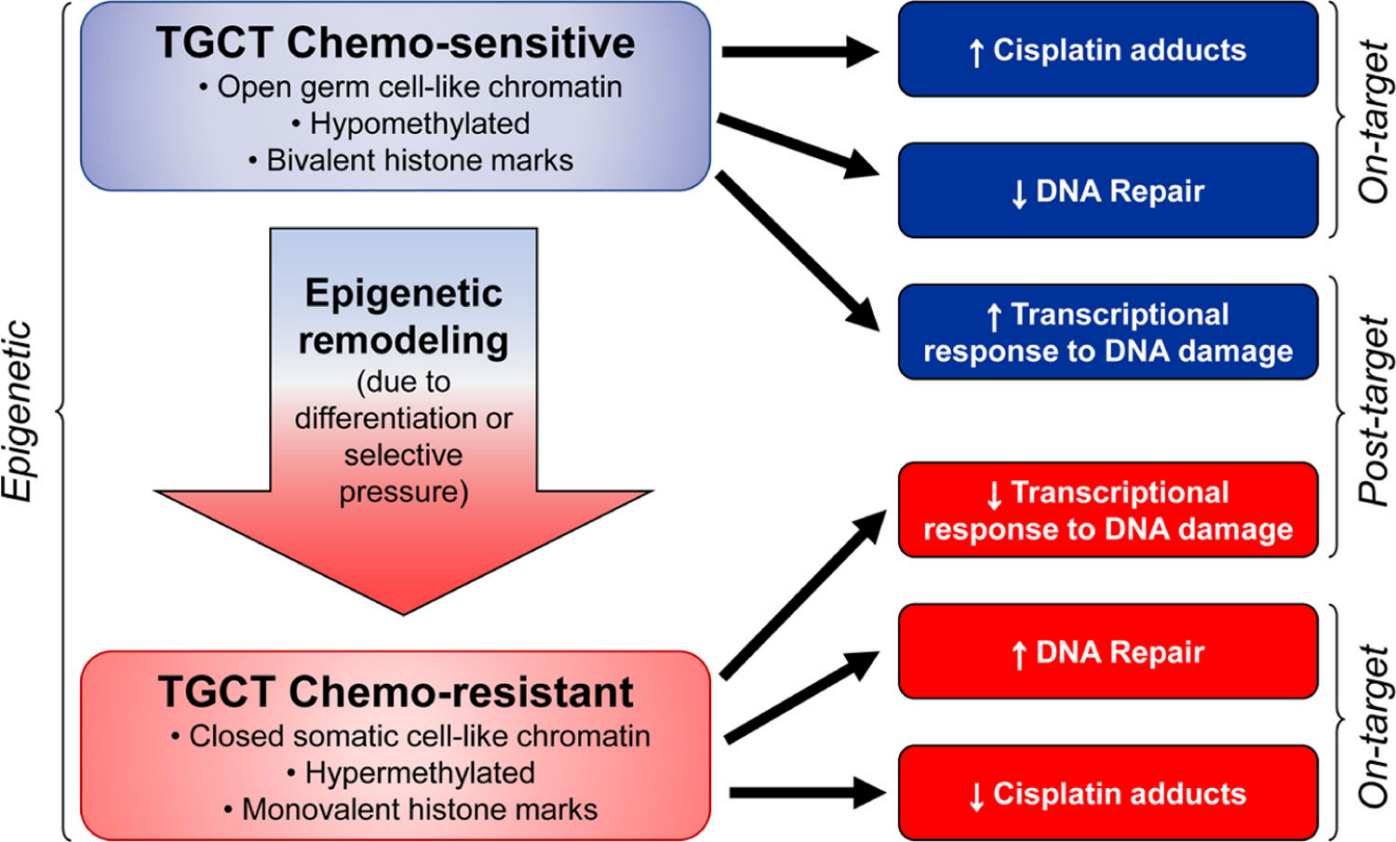
Epigenetic context as a key determinant of cisplatin sensitivity and resistance in TGCTs. TGCTs: testicular germ cell tumors

We speculate that epigenetic context is a key driver of cisplatin resistance and sensitivity in TGCTs and specifically the DNA hypomethylation may be associated with cisplatin hypersensitivity [Figure 5]. This could be achieved by at least two, mutually-nonexclusive, mechanisms. In the first (on-target) more “open”, pluripotent-like chromatin may be more accessible to cisplatin binding and adduct formation. In the second (post-target/off target) the open, pluripotent chromatin of TGCTs may possess an inherent transcriptional plasticity not shared with other solid cancers. This may allow for a more robust acute transcriptional response to DNA damage, for example mediated by p53. In this model, epigenetic reprogramming associated with loss of pluripotency would be a source of cisplatin resistance in teratomas and perhaps other TGCT subtypes than are or become refractory to cisplatin [Figure 5]. A more complete understanding of the epigenetic state of sensitive and resistant TGCTs, especially in the clinical setting is needed to fully test this model.

## CONCLUSIONS

Mechanisms of cisplatin hypersensitivity and acquired resistance in TGCTs are likely multi-factorial and associated with the germ cell origins of these solid tumors. Epigenetics may play a more prominent role in the biology of TGCTs compared to somatic solid tumors that possess higher mutation rates and more frequent and obvious driver mutations. This likely extends to one of the important clinical features of TGCT, their curability. Considering that epigenetic states are malleable, one can envision reinstating “sensitive” epigenetics to cisplatin refractory TGCTs and theoretically even to common somatic solid tumors. Of course and as always, how these strategies alter toxicity to normal tissues will be an important issue.
